# Supplementary material for: MetaRibo-Seq measures translation in microbiomes
Source: Nat Commun. 2020 Jun 29;11:3268. doi: 10.1038/s41467-020-17081-z (PMC7324362; doi:10.1038/s41467-020-17081-z)
Supplement: Supplementary file 10 — Supplementary Data 7 [file 41467_2020_17081_MOESM10_ESM.zip › File2/Confidence_High_Taxonomy/32883_out.krona.html]

Javascript must be enabled to view this page.

members
magnitude
magnitudeUnassigned
count
unassigned
taxon
rank

32883\_out


SRS018623\_contig\_number\_contig-100\_809.81539SRS077294\_contig\_number\_11455SRS077589\_contig\_number\_contig-100\_2807.2808SRS1055083\_contig\_number\_contig-100\_1746.37071SRS893252\_contig\_number\_contig-100\_23531.23532
31
5

superkingdom
26
2

1
976
phylum

class
200643
1

171549
1
order

171550
1
family

1
239759
genus

1

SRS015431\_contig\_number\_56046
214856
species

1239
25
phylum

class
25
186801

order

SRS044535\_contig\_number\_contig-100\_389.147320
186802
25
1

family
541000
24

genus
20
216851

SRS063370\_contig\_number\_contig-100\_1117.58471
1

19
853

SRS013965\_contig\_number\_39929SRS015663\_contig\_number\_23424SRS018817\_contig\_number\_20101SRS024435\_contig\_number\_43823SRS043411\_contig\_number\_2718SRS045195\_contig\_number\_6998SRS047433\_contig\_number\_contig-100\_396.109827SRS049712\_contig\_number\_contig-100\_251.69016SRS063518\_contig\_number\_39739SRS064757\_contig\_number\_23566SRS076756\_contig\_number\_10557SRS098881\_contig\_number\_23001SRS1041164\_contig\_number\_4887SRS143417\_contig\_number\_28447SRS143876\_contig\_number\_13384SRS144362\_contig\_number\_43200SRS144506\_contig\_number\_47891SRS147557\_contig\_number\_33563SRS148874\_contig\_number\_6029
species

genus
4
946234

4

SRS015486\_contig\_number\_1021SRS019068\_contig\_number\_109688SRS043841\_contig\_number\_6835SRS078419\_contig\_number\_contig-100\_1581.198454
1193534
species
